# Galectin-8 Immunohistochemical Profile in Pancreatic Ductal Adenocarcinoma: Emerging Evidence for Its Prognostic Role

**DOI:** 10.3390/diagnostics13203215

**Published:** 2023-10-15

**Authors:** Andreea Rusu, Irina-Draga Caruntu, Ludmila Lozneanu, Delia Gabriela Ciobanu, Cornelia Amalinei, Simona-Eliza Giusca

**Affiliations:** 1 Department of Morpho-Functional Sciences I—Histology, “Grigore T. Popa” University of Medicine and Pharmacy, 700115 Iași, Romania; andreeadima27@yahoo.com (A.R.); ludmila.lozneanu@umfiasi.ro (L.L.); cornelia.amalinei@umfiasi.ro (C.A.); 2 Department of Pathology, “Dr. C.I. Parhon” Clinical Hospital, 700503 Iasi, Romania; 3 Department of Pathology, “Sf. Spiridon” Clinical Emergency County Hospital, 700111 Iasi, Romania; delia.ciobanu@umfiasi.ro; 4 Department of Morpho-Functional Sciences I—Morphopathology, “Grigore T. Popa” University of Medicine and Pharmacy, 700115 Iași, Romania

**Keywords:** galectin-8, pancreatic ductal adenocarcinoma, immunohistochemistry, histological pattern, prognosis

## Abstract

Pancreatic ductal adenocarcinoma (PDAC) represents the most frequent pancreatic malignancy, with stromal and epithelial heterogeneity reflected in outcome variability. Therefore, a molecular classification is promoted based on the validation of new diagnostic and prognostic markers. Galectin-8 (Gal8) has been pointed out as a prognostic factor for survival in several types of tumors. Due to limited existing data on PDAC, our study aimed to evaluate the Gal8 profile in PDAC alongside its prognostic status. A total of 87 cases of PDAC were immunohistochemically investigated, and Gal8 immunoexpression was qualitatively and semi-quantitatively assessed and correlated with classical clinicopathological parameters and survival. Gal8 immunoexpression was identified to be mostly nuclear and cytoplasmic, followed by exclusively cytoplasmic and exclusively nuclear. A statistical analysis between Gal8 profiles defined by negative, low, or high scores and clinicopathological characteristics showed significant differences in tumor size, pN stage, and lympho-vascular invasion. Although a Cox regression analysis did not support the prognostic status of Gal8, and we did not confirm its relationship with OS, our results show that exclusively nuclear labeling was associated with an increased mean OS compared with cytoplasmic and nuclear labeling (29.37 vs. 17.93 months). To the best of our knowledge, this is the first study to report a detailed pattern of Gal8 immunostaining in PDAC and to correlate this pattern with clinicopathological characteristics and survival. Our results show that Gal8 immunoexpression is associated with a more aggressive phenotype, thus opening perspectives for larger studies to validate Gal8 as a prognostic factor.

## 1. Introduction

Pancreatic ductal adenocarcinoma (PDAC) consists of a spectrum of pancreato-biliary phenotype proliferations, at the moment with a minimally improved prognosis [1,2,3]. The unfavorable prognosis indicated by high mortality rates [4] is mainly due to a diagnosis in advanced stages that leads to only 10–20% of cases being suitable for surgical resection, making the majority of PDAC patients an oncological challenge because of the low response to available treatment options [3,4]. The hallmarks of PDAC are epithelial compartment genetics and morphological heterogeneity alongside a stromal compartment that represents the main tumor bulk [5]. Although there has been a trend to dichotomize PDAC based on molecular features and the different responses to adjuvant therapy, namely, “classical” and “basal-like” PDAC, currently, the PDAC diagnosis is based on classic microscopic features and prognostic elements that fail to stratify and predict the outcome of patients [3,6,7,8]. Thus, two pattern-based morphological classification of PDAC have been proposed, which correlates molecular features with morphology and clinical outcomes [2,9].

Galectins have emerged as a family of proteins with numerous members involved in different stages of the carcinogenic process as mediators of tumor growth and metastatic spread [10,11].

Galectin-8 (Gal8), similar to other galectins, has multiple compartment positions. It is secreted as a cytoplasmic protein with carbohydrate-independent binding activities and, after non-classical secretion, presents extracellular carbohydrate-dependent activity [12]. The loop can be closed through the endocytic route, through its reentering of the intracellular compartment, or it can be trapped extracellularly due to glycan–lectin interactions [12,13]. The molecular structure and cellular position of Gal8 allow it to modulate cell signaling, migration, and adhesion [13,14,15], being involved in tumor-promoting inflammation and angiogenesis [10].

Gal8 has been studied in various tumors from the digestive and reproductive systems [13,16,17,18]. In the digestive system, a high immunohistochemical (IHC) expression of Gal8 has been correlated with tumor growth rate for colonic malignant tissues [16] and has been pointed to as a good prognostic factor due to its statistically significant associations with overall survival (OS) and disease-free survival (DFS) in gastric cancer [18]. In the reproductive system, high Gal8 immunoexpression was also found to be a good prognostic factor for cervical carcinoma, being significantly statistically associated with the histological subtype, a lower tumor stage, a negative lymph node status, and relapse-free survival (RFS) [15]. Additionally, the Gal8 nuclear immunoreaction was associated with better DFS and OS in triple-negative breast cancer, despite the absence of any clinicopathological associations [19]. Moreover, in breast cancer, the Gal8 interaction with the activated leucocyte cell adhesion molecule (ALCAM), its endothelial ligand, was identified as a mechanism responsible for ALCAM surface segregation, possibly preventing its internalization and, consequently, accelerating tumor cell spread [13].

Limited data on Gal8 immunoexpression and its relationship with pancreatic carcinogenesis are available [20], although other galectin family members have been studied and connected to pancreatic stellate cell activation (Gal1 and Gal3); the modulation of the immune response (Gal1, Gal3, and Gal9); and the inducer (Gal1) or inhibitor (Gal4) of tumor cell proliferation, invasion, and migration [11].

To the best of our knowledge, the value of Gal8 expression as a prognostic factor in PDAC has not been previously reported.

Within this context, our study focused on the immune profile of Gal8 in primary PDAC, aiming to identify the peculiar aspect of its expression in correlation with clinicopathological and survival parameters and to assess its potential as a prognostic factor.

## 2. Materials and Methods

### 2.1. Patients and Tissue Samples

This study was conducted on archived paraffin-embedded formalin-fixed tissues obtained from 87 patients diagnosed between 2007 and 2017 with malignant pancreatic ductal proliferation as follows: PDAC NOS in 85 cases and adenosquamous carcinomas (with an extensive ductal component) in 2 cases. Because of the extensive ductal component, all 87 cases were analyzed as a unitary group. 

The inclusion criteria were surgery without previous chemotherapy, a clear pancreatic ductal origin, and available clinicopathological and survival data. The clinicopathological data were collected from patients’ medical records. The patients were followed between a minimum of 24 months and a maximum of 120 months. The research was approved by the Ethics Committee of “Sf. Spiridon” Clinic Emergency County Hospital of Iasi and the Ethics and Research Committee of “Grigore T. Popa” University of Medicine and Pharmacy, Iasi (no. 59700/27 November 2015, no 1/1 September 2017). 

All cases were reassessed and staged in agreement with the 8^th^ edition of the TNM Classification of Malignant Tumors [6] and the 5th edition of Digestive System Tumors [3]. We also evaluated the associated preneoplastic lesions; the histopathological patterns, including the percentage held in tumor bulk [2,9]; and the ratio between metastatic lymph nodes and harvested lymph nodes (LNR) [21]. The evaluation was independently performed by 2 pathologists, and differences were solved through consensus.

### 2.2. Immunohistochemical Exam

For each case, paraffin-embedded blocks were selected for an IHC exam based on the morphological aspects identified in the microscopic specimens, relevant for primary PDAC morphology or patterns and associated lymph node metastases. 

In short, 4 µm thick tissue sections were sliced from each paraffin block, dewaxed via complete immersion in xylene (3 h at 58 °C and 10 min at room temperature), and rehydrated via immersion in 4 successive alcohol baths (100%, 90%, 80%, and 70%). The heat-induced antigen retrieval method was chosen for unmasking antigenic epitopes, using an epitope retrieval solution with pH 9 for 30 min (Leica Biosystems, Weitzlar, Germany). The slides were treated with 3% hydrogen peroxide to block endogenous peroxidase activity. The incubation of the primary antibody, anti-galectin-8 (rabbit monoclonal, ab109519, dilution 1/250, Abcam, Cambridge, MA, USA), was performed overnight at 4 °C. The primary antibody reaction was amplified using a compatible polymer detection system (ab64261, Abcam, Cambridge, MA, USA), and immunolabeling was visualized using a 3.3′-diaminobenzidine tetrahydrochloride chromogen. For technique control, we used pancreatic tissue as an internal positive control [17] or prostatic adenocarcinoma tissue as an external positive control. Negative control was achieved by omitting the primary antibody.

### 2.3. Qualitative and Semi-Quantitative Evaluation of Gal8 

IHC labeling was evaluated qualitatively, taking into account the subcellular location of Gal8 in relation with histopathological patterns, and it was evaluated semi-quantitatively based on the percentage of positive cells and the intensity of the staining. We applied a scoring system proposed for gastric adenocarcinoma [18]. There were 4 classes of immunolabeling intensity (I), namely, 0 = unstained/negative; 1 = weak; 2 = moderate; and 3 = strong (the same as Langerhans Isle immunostaining). The slides were analyzed in their entirety, and, in the case of labeling intensity variability, the dominant intensity was chosen. As far as the percentage of immunolabeling (P) is concerned, the results were registered as factual percentages. The final immunoscore was obtained by multiplying I by P, with a score range between 0 and 300. After a score distribution analysis, the cut-off was calculated by adding up the median and interquartile ranges. Based on the established cut-off, the study group was divided into three classes: 0 = negative score, 1–192 = low score, and >192 = high score. 

### 2.4. Statistical Analysis

The data were recorded as absolute value/percentages or median/ranges. A statistical analysis was performed using SPSS version 20 (IBM, Armonk, NY, USA) and Microsoft Excel 2016 (Microsoft, Redmond, WA, USA). The significance of the association between the clinicopathological parameters and the Gal8 immunoscore was determined using Pearson’s chi-square and Fisher’s exact tests. A receiver operating characteristic (ROC) curve was applied to assess the cut-off point for the LNR. A survival analysis based on Kaplan–Meyer curves used the log-rank test (Mantel–Cox) to compare the survival rate between the studied groups, and the prognostic significance of clinicopathological parameters was evaluated through a univariate Cox regression.

## 3. Results

### 3.1. Clinicopathological Characteristics

The main clinicopathological characteristics of the study group are summarized in Table 1.

Based on gender, the patients were divided almost equally between males (41 cases—47.13%) and females (46 cases—52.87%), and they had a mean age of 60.77 years (ranging from 35 to 79). Twelve patients were diagnosed before the age of 50, and 19 patients were diagnosed after the age of 70. 

In 19 cases (21.83%), PDAC was directly connected with a preneoplastic lesion as follows: intraductal papillary mucinous neoplasms (IPMNs) in 13 cases (14.94%), a mucinous cystic neoplasm (MCN) in 3 cases (3.44%), an intraductal oncocytic papillary neoplasm (IOPN) in 2 cases (2.29%), and an intraductal tubulopapillary neoplasm (ITPN) in 1 case (1.14%). In six cases (6.89%), there was also a concomitant pancreatic intraepithelial neoplasia (PanIn) in the PDAC-adjacent areas, either high-grade or low-grade. 

Following the tumoral component, 57 cases (65.51%) were classified as conventional PDAC and 30 cases (34.48%) as non-conventional PDAC. Based on the histological pattern, 50 cases (57.47%) were classified as having a glandular (gland-forming) pattern and 38 cases (43.67%) as having a non-glandular (non-gland-forming) pattern. According to the number of tumoral patterns found in a case, 40 tumors (45.97%) showed a homogeneous morphology with one dominant pattern, while 47 tumors (54.03%) were heterogeneous, with 33 cases (37.93%) presenting two patterns and 14 cases (16.09%) having three patterns. Thus, in the 87 cases included in the study group, we identified 148 tumoral patterns—the most frequent being the common one (75 cases), followed by foamy cell (23 cases), cribriform (18 cases), large-duct (12 cases), basal/composite area (13 cases), and micropapillary (7 cases) patterns. 

In our study group, around two-thirds of the patients had tumors smaller than 4 cm (55 cases—63.21%), and one-third had large tumors (32 cases—36.78%), being staged as pT1 in 9 cases (10.34%), pT2 in 46 cases (52.87%), pT3 in 30 cases (34.48%), and pT4 in 2 cases (2.29%). In 24 cases (27.58%), lymph node metastases were absent (pN0). The 63 patients (72.41%) with lymph node metastases presented mainly one to three positive lymph nodes (43 cases—49.42%), staged as pN1, with more than four positive lymph nodes in a minority of cases (20 cases—22.98%), staged as pN2. The median value of LNR was 0.13. Based on this ratio, 55 cases (63.21%) were above the 0.08 cut-off value, and 32 cases (36.78%) were under it. Subsequently, taking into account the tumor dimension and lymph node metastases, patients were grouped as low-stage in 23 cases (26.43%) and high-stage in 64 cases (73.56%).

The evaluation of lympho-vascular and perineural tumoral extension revealed that tumor cells were present in the vessels in 60 cases (68.96%) and in the nerves in 81 cases (93.10%), meaning that most patients were LV1 and Pn1.

The mean OS of the study group was 18.92 months (ranging from 1 to 84 months), with a median of 14 months. In January 2022, 7 patients (8.04%) were alive, and 80 patients (91.95%) had died. The living group had a mean survival rate of 48.42 months (ranging from 16 to 84 months). Among the deceased patients, 66 (75.86%) died in the first two years after their diagnosis, distributed as follows: 8 in the first month after surgery, 28 in the first year, and 30 in the second year; the 5-year OS was 5.74%.

### 3.2. Qualitative Assessment of Gal8 Immunoexpression 

The PDAC qualitative assessment of Gal8 immunostaining revealed a positive reaction in 67 cases (77%) and an unstained/negative one in 20 cases (23%). The associated preneoplastic lesions showed a similar Gal8 immunoreaction (either positive or negative) to the invasive malignant proliferations. The normal adjacent pancreatic parenchyma was positive for Gal8. 

For the 67 cases with a Gal8 positive immunoreaction, there were different subcellular localizations: mostly nuclear and cytoplasmic in 40 cases (59.70%), followed by exclusively cytoplasmic in 19 cases (28.35%) and exclusively nuclear in 8 cases (11.94%). We also noted that, in three cases with an evident sequence from the ductal epithelium with low-grade–high-grade features to invasive carcinoma, there was a switch of Gal8 labeling from the cytoplasmic compartment in the intraductal epithelium to nuclear labeling in invasive glands.

**Figure 1:**
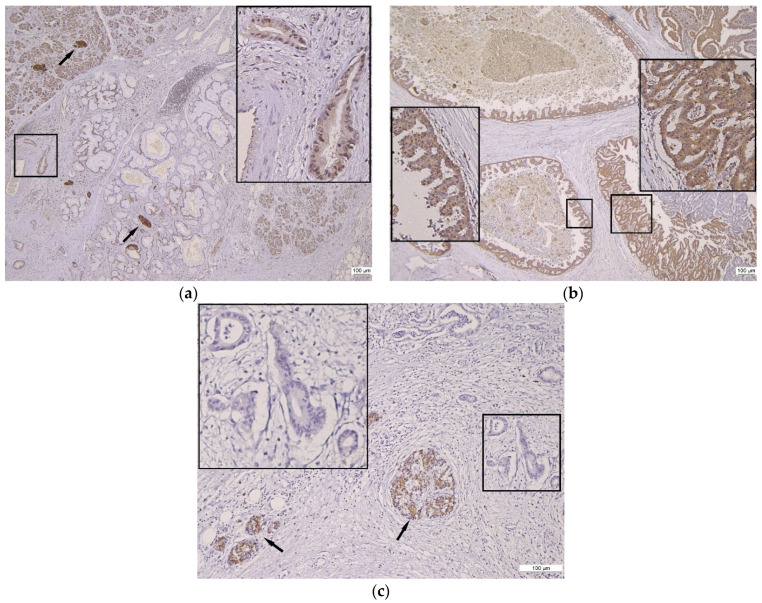
Gal8 immunostaining in pancreatic tissue and ductal proliferation spectrum. (**a**) Gal8 positive reaction in normal pancreatic parenchyma, PanIn, and PDAC, 4×; insert: PDAC with weak cytoplasmic and moderate nuclear Gal8 staining, 20×; arrows: internal control—strong intensity of Gal8 staining in Langerhans Isle. (**b**) Heterogeneous Gal8 positive reaction in IPMN, 4×; inserts: moderate cytoplasmic and nuclear Gal8 staining (left), strong cytoplasmic and nuclear Gal8 staining (right). (**c**) Negative Gal8 immunoreaction in PDAC, 10×; insert: unstained/negative PDAC glands; arrows: internal control—strong intensity of Gal8 staining in Langerhans Isle.

**Figure 2:**
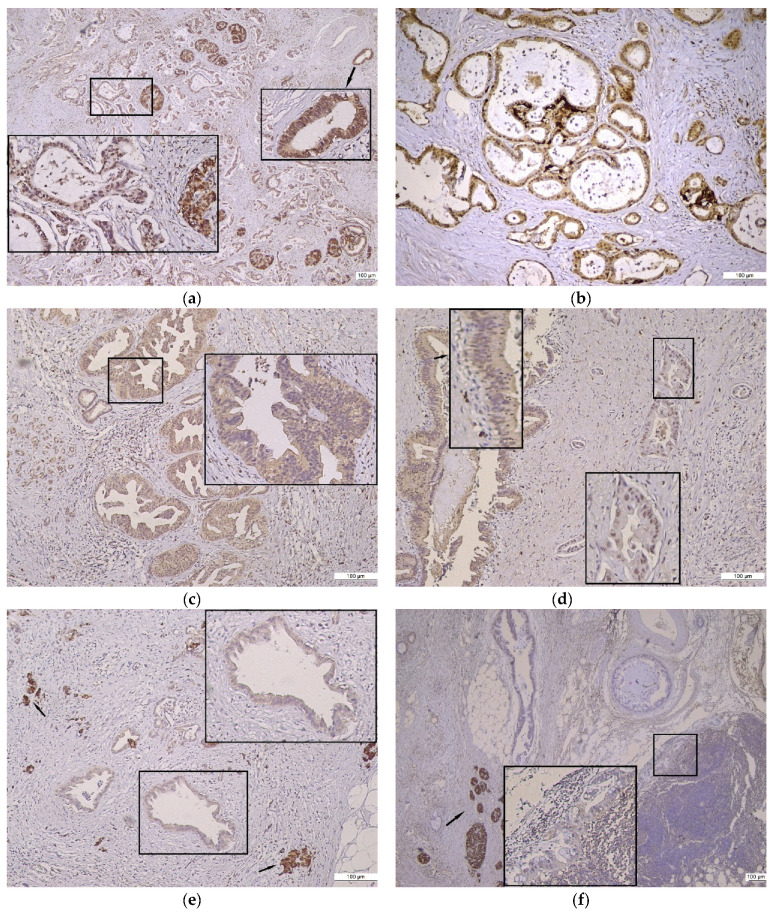
Gal8 positive immunoreaction in PDAC. (**a**) Strong Gal8 staining in heterogeneous PDAC, 4×; inserts: moderate cytoplasmic and strong nuclear Gal8 staining (left), strong cytoplasmic and nuclear Gal8 staining (right). (**b**) Moderate cytoplasmic and strong nuclear Gal8 staining in homogeneous conventional PDAC, 10×. (**c**) PDAC Gal8 immunostaining with moderate intensity, 10×; insert: exclusively cytoplasmic immunoreaction. (**d**) Conventional PDAC Gal8 immunostaining with moderate intensity, 10×; inserts: exclusively cytoplasmic immunoreaction in intraductal epithelium (left), exclusively nuclear immunoreaction in PDAC (right). (**e**) PDAC Gal8 immunostaining with weak intensity, 10×; insert: exclusively cytoplasmic immunoreaction in PDAC; arrows: internal control—strong intensity of Gal8 staining in Langerhans Isle. (**f**) Gal8 immunostaining with weak intensity, 10×; insert: PDAC subcapsular metastasis in lymph node—exclusively cytoplasmic immunoreaction; arrow: internal control—strong intensity of Gal8 staining in Langerhans Isle.

The heterogeneity of Gal8 intensity and the subcellular localization in normal tissue, preneoplastic lesions, and PDAC are illustrated in Figure 1 and Figure 2. 

The analysis of the Gal8 subcellular localization in tandem with the morphological pattern revealed several heterogeneous aspects, as detailed below.

The dual Gal8 immunostaining, nuclear and cytoplasmic, was found predominantly in conventional or glandular PDAC with a common pattern and a homogenous morphology dominated by a single pattern (22 out of 40 cases). The remaining 18 cases with dual immunostaining had foamy cell, cribriform, micropapillary, large-duct, and poorly differentiated areas with basal/composite patterns of PDAC. 

The exclusively nuclear staining was also found predominantly in conventional or glandular PDAC with a common pattern and increased heterogeneity associating other patterns within the same tumor (six out of eight cases). The other two cases with nuclear labeling had large-duct and foamy cell patterns.

The exclusively cytoplasmic staining was distributed in all tumor patterns almost equally. The large-duct, micropapillary, and basal/composite areas were always Gal8 positive.

Gal8 immunoexpression was absent only in the common, foamy cell, and cribriform patterns. 

Of the 26 cases analyzed for Gal8 immunoexpression in lymph node metastases, 11 (42.30%) were Gal8 positive, and 15 (57.69%) were Gal8 negative. Compared to Gal8 in the primary neoplastic process, 16 cases (61.53%) showed lower Gal8 immunostaining, while in the other 10 cases (38.46%), the Gal8 expression was similar (7 cases were Gal8 negative, and 3 cases were Gal8 positive). 

### 3.3. Semi-Quantitative Assessment of Gal8 Immunoexpression

The semi-quantitative Gal8 evaluation revealed 20 cases (22.98%) with a negative score, 47 cases (54.02%) with a low score, and 20 cases (22.98%) with a high score.

The detailed results of the semi-quantitative Gal8 profile in relation to the main clinicopathological characteristics are summarized in Table 1.

The ranking of classes with a negative, a low, and a high Gal8 score, in relation to the clinicopathological characteristics, revealed the following differences: the female gender showed mainly a negative and low Gal8 expression, and the patients with a tumor size of less than 4 cm, in the T2 stage, and with a glandular pattern showed predominantly low and high Gal8 expression. The patients with a tumor size greater than 4 cm and in the T3 stage had mainly negative Gal8 expression.

As far as all the other clinicopathological features are concerned, in all three subgroups defined by system scoring, most cases were aged 51 to 70, and they were characterized by a high TNM stage, G2 differentiation, lympho-vascular and perineural invasion, and a conventional morphology.

### 3.4. Correlation between Gal8 Immunoexpression and Clinicopathological Characteristics

The statistical analysis between the Gal8 immunohistochemical profile, defined by negative, low, and high scores, and the clinicopathological characteristics revealed significant differences in tumor size (*p* = 0.01), pN stage (*p* = 0.04), and lympho-vascular invasion (*p* = 0.03) (Table 1).

No statistically significant differences were detected for the other clinicopathological characteristics.

### 3.5. Correlation between Gal8 Immunoexpression and Survival

The survival analysis correlated with the Gal8 score revealed a mean OS of 17.55 months for negative Gal8, 18.70 months for low Gal8, and 21.10 months for high Gal8. In terms of different subcellular localization, regardless of the overall score, there was also a variability in the OS results. Concretely, for the exclusively cytoplasmic staining group, the mean OS was 21.57 months; for the exclusively nuclear staining group, it was 29.37 months; and for the dual staining, cytoplasmic and nuclear group, it was 17.93 months.

**Figure 3:**
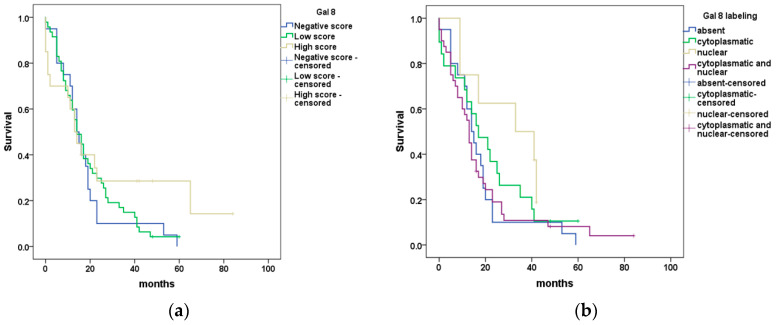
Kaplan–Meier curves showing (**a**) differences in OS for PDAC patients stratified using Gal8 immunoscore; (**b**) differences in OS for PDAC patients stratified using Gal8 subcellular localization.

The Kaplan–Meier curves and log-rank test revealed the absence of statistically significant differences between patient survival and Gal8 semi-quantitative evaluation or subcellular localization (*p* = 0.39 and *p* = 0.22, respectively) (Figure 3a,b).

**Figure 4:**
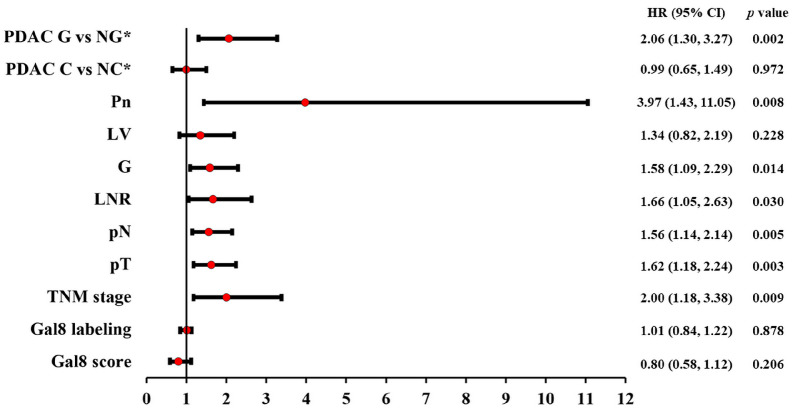
Univariate Cox regression results: Forest plot with hazard ratio (HR) and 95% confidence interval (CI) for microscopic prognostic factors in PDAC. * G—glandular; NG—non-glandular; C—conventional; NC—non-conventional.

The univariate Cox regression confirmed the prognostic factor status only for the pTNM stage, grouped and single (pT and pN stages); tumor grade (G); perineural invasion (Pn); lymph node ratio (LNR); and morphological classification of glandular vs. non-glandular pattern. Lympho-vascular invasion and the morphological classification of conventional vs. non-conventional PDAC were not confirmed as prognostic factors (Figure 4).

Also, the univariate Cox regression did not support a prognostic factor status in PDAC for Gal8 IHC expression, both for subcellular labeling (*p* = 0.87, HR = 1.01) and for the general immunoscore (*p* = 0.20, HR = 0.80) (Figure 4).

## 4. Discussion

Since it was first described [22], Gal8, a tandem-repeat-type galectin with two carbohydrate recognition domains connected by a small peptide region, has been reported to be widely expressed in numerous normal and tumor tissues [20,23]. Moreover, Gal8 has extended localization in subcellular compartments (nuclear, cytoplasmic, and membrane compartments), as well as in extracellular compartments, which allows the molecule to bind to different ligands [13,24]. Gal8 immunohistochemical expression, as a prognostic factor of survival, has been reported in gastric cancer [18], breast cancer [19], ovarian cancer [25], and cervical cancer [15].

To the best of our knowledge, this is the first study to report a detailed pattern of Gal8 immunostaining, to correlate this pattern with clinicopathological characteristics and survival, and to analyze its potential as a “candidate” prognostic factor.

In our cohort, Gal8 staining was found in the tumor core and associated preneoplastic lesions, and only in the subcellular compartment—either in the cytoplasmic and nuclear compartments, or exclusively in the cytoplasmic or nuclear compartment—without membrane or stromal staining identified in other galectin family members [26,27]. 

This heterogeneity was complemented by the identification of Gal8 labeling translocation from the cytoplasm, in the intraductal epithelium with low-grade–high-grade lesions, to nuclei, in tumor invasive glands. It is worth noting that we also demonstrated Gal8 immunoexpression in PDAC lymph node metastases, with a profile similar to or different from that of the primary tumor. All these observations may indicate different stages of cellular transformation in the process of carcinogenesis and metastasis, which may be translated into prognostic assessment and, possibly, into therapeutic targets.

We found that both high Gal8 immunoexpression and low Gal8 immunoexpression correlate with tumors smaller than 4 cm, but high Gal8 is associated with an increased number of positive lymph nodes and lympho-vascular invasion, while low Gal8 corresponds to a lower number of positive lymph nodes and tumor emboli. However, the Gal8 negative status was correlated with tumors larger than 4 cm and with the highest number of lymph node metastases and lympho-vascular invasion. These results suggest that Gal8 immunoexpression leads to a more aggressive phenotype with pro-metastatic features from the early stages, although tumor cell proliferation per se is not reflected in an increased tumor size.

The mechanism used by Gal8 to generate a smaller PDAC with a larger number of metastasized lymph nodes may be the interaction with its endothelial cell ligand CD166 [28]. Moreover, CD166 has already been established as a prognostic factor in PDAC [29,30,31,32]. Beside endothelial cell interaction, Gal8—CD166 have been described in experimental studies using breast cancer cells [33] and cervix carcinoma cells [34]. It is worth mentioning that CD166, also known as ALCAM, is a transmembrane glycoprotein from the immunoglobulin superfamily involved in PDAC cells’ proliferative and migratory properties [30], chemoresistance [35], vascular dissemination [32], and interactions with pancreatic stellate cells [36]. CD166 availability is modulated by Gal8 [34,37], and a study of breast cancer cells revealed a relevant association between tumor dimension and these two ligands [33].

Although we did not find Gal8 expression to be associated with OS, our results are worth discussing in regard to the immunostaining localization. The Gal8 subcellular localization has been associated with different results in breast cancer patient survival, with nuclear Gal8 being correlated with a significantly better DFS in triple-negative breast cancer, while cytoplasmic staining has been associated with a better outcome in no special type tumors [19,38]. Similarly to breast cancer data, in our study, PDAC with nuclear labeling had an increased mean OS compared to tumors with cytoplasmic and nuclear labeling (29.37 vs. 17.93 months), although without significant OS differences based on the staining pattern or overall Gal8 immunohistochemical score.

These results can be explained by the main limitation of our study, namely, the relatively small number of patients compared with Gal8 studies on breast, colon, or gastric cancer, a difference that derives from the incidence of pancreatic cancer being lower than that of the above-mentioned neoplasia, as well as from the lower percentage of patients that undergo surgery. Another limitation stems from the fact that this study was focused on the histopathological features, without integrating an assessment of tumor serological markers, biological profile, and/or imaging data.

Within this context, our results can be considered a starting point for further research on the relationship between Gal8 immunoexpression and survival parameters in PDAC, taking into account that the undisputable confirmation of the prognostic value of Gal8 must be supported by investigating a larger number of cases.

Based on the molecular structure and subcellular position, Gal8 has been linked in tumor biology with the modulation of cell adhesion and metastatic capacity [14,16,17,39,40], as well as with endocytosis [34], angiogenesis [14,28,41], and the immune response [42,43,44]. However, there are very little data on its involvement in pancreatic carcinogenesis. Therefore, we can summarize the following learning objectives resulting from our study:

- The histopathological profile of PDAC is complex, and numerous morphological variants have been described, yet this variability cannot currently be directly associated with tumor behavior;
- The molecular profile of PDAC is incompletely defined; we do not have, at this time, confirmed molecular prognostic factors;
- Similarly to breast, colon, gastric, cervical, and prostatic cancer, the Gal8 heterogeneity in PDAC requires its investigation in correlation with the clinicopathological characteristics and survival parameters, aiming to confirm its potential prognostic value.

Our results confirm correlations between heterogeneous Gal8 immunoexpression and several clinicopathological characteristics (tumor size, lympho-vascular invasion, and lymph node metastasis), meaning that different tissue levels of Gal8 could lead to different tumor behaviors. Thus, a more aggressive phenotype for PDAC may be revealed by further studies that also include Gal8 as a potential prognostic factor.

## Figures and Tables

**Table 1:** Clinicopathological characteristics and Gal8 immunoscore of PDAC patients.

Clinicopathological Characteristics	Gal8 Negative Score	Gal8 Low Score	Gal8 High Score	*p* Value
Gender				
Female	12 (60%)	26 (55.32%)	8 (40%)	0.39
Male	8 (40%)	21 (44.68%)	12 (60%)	
Age				
≤50 years	3 (15%)	6 (12.77%)	3 (15%)	
51–70 years	14 (70%)	30 (63.83%)	12 (60%)	0.94
>70 years	3 (15%)	11 (23.40%)	5 (25%)	
Tumor size				
≤4 cm	8 (40%)	30 (63.82%)	17 (85%)	0.01
>4 cm	12 (60%)	17 (36.17%)	3 (15%)	
T stage				
T1	1 (5%)	5 (10.63%)	3 (15%)	
T2	7 (35%)	25 (53.19%)	14 (70%)	0.07
T3	11 (55%)	17 (36.17%)	2 (10%)	
T4	1 (5%)	0 (0%)	1 (5%)	
N stage				
N0	5 (25%)	16 (34.04%)	3 (15%)	
N1	8 (40%)	26 (55.31%)	9 (45%)	0.04
N2	7 (35%)	5 (10.63%)	8 (40%)	
Lymph node ratio				
LNR ≤ 0.08	15 (75%)	25 (53.20%)	15 (75%)	0.44
LNR > 0.08	5 (25%)	22 (46.80%)	5 (25%)	
TNM stage				
Low (Ia, Ib, IIa)	4 (25%)	16 (34.04%)	3 (15%)	0.20
High (IIb, III, IV)	16 (75%)	31 (65.96%)	17 (85%)	
Tumor grade				
G1	6 (30%)	12 (25.53%)	2 (10%)	
G2	10 (50%)	32 (68.09%)	17 (85%)	0.13
G3	4 (20%)	3 (6.38%)	1 (5%)	
Lympho-vascular invasion				
LV0	4 (20%)	20 (42.55%)	3 (15%)	0.03
LV1	16 (80%)	27 (57.44%)	17 (85%)	
Perineural invasion				
Pn0	0 (0%)	4 (8.5%)	2 (10%)	0.53
Pn1	20 (100%)	43 (91.5%)	18 (90%)	
Pattern-based classification				
Conventional PDAC	12 (60%)	31 (65.95%)	14 (70%)	0.79
Non-conventional PDAC	8 (40%)	16 (34.05%)	6 (30%)	
Glandular PDAC	10 (50%)	26 (55.32%)	13 (65%)	0.61
Non-glandular PDAC	10 (50%)	21 (44.68%)	7 (35%)	

## Data Availability

The data used to support the findings of this research are available upon request to the authors.
